# The Liberation of Embryonic Stem Cells

**DOI:** 10.1371/journal.pgen.1002019

**Published:** 2011-04-07

**Authors:** Kathryn Blair, Jason Wray, Austin Smith

**Affiliations:** Wellcome Trust Centre for Stem Cell Research, University of Cambridge, Cambridge, United Kingdom; Harvard Medical School, United States of America

## Abstract

Mouse embryonic stem (ES) cells are defined by their capacity to self-renew and their ability to differentiate into all adult tissues including the germ line. Along with efficient clonal propagation, these properties have made them an unparalleled tool for manipulation of the mouse genome. Traditionally, mouse ES (mES) cells have been isolated and cultured in complex, poorly defined conditions that only permit efficient derivation from the 129 mouse strain; genuine ES cells have not been isolated from another species in these conditions. Recently, use of small molecule inhibitors of glycogen synthase kinase 3 (Gsk3) and the Fgf-MAPK signaling cascade has permitted efficient derivation of ES cells from all tested mouse strains. Subsequently, the first verified ES cells were established from a non-mouse species, *Rattus norvegicus*. Here, we summarize the advances in our understanding of the signaling pathways regulating mES cell self-renewal that led to the first derivation of rat ES cells and highlight the new opportunities presented for transgenic modeling on diverse genetic backgrounds. We also comment on the implications of this work for our understanding of pluripotent stem cells across mammalian species.

## Introduction

Embryonic stem (ES) cells were first isolated in 1981 by Martin in California [1] and Evans and Kaufman in Cambridge [2]. These cells derive from the transient epiblast compartment of the pre-implantation mouse blastocyst that would go on to form the embryo proper in vivo [3]. In vitro, ES cells can self-renew indefinitely without genetic transformation, can be expanded clonally, and retain pluripotency, which is the ability to differentiate into all adult cell types, including the germ cells [4]. The development of homologous recombination technology in cultured mammalian cells and its application to mouse ES (mES) cells made possible extensive targeted manipulation of the mouse genome; the engineered cell lines and the mice derived from them have revolutionized our ability to study the effects of gene function in mammalian biology and disease [5]. In 2007, the importance of these technological advances was recognized by the Nobel Committee, who awarded the Prize in Physiology or Medicine to Evans, Capecchi, and Smithies [6].

Hopes that other animals would yield ES cells, facilitating genetic manipulation in diverse species, met with frustration [7]. While cell lines could be established from early embryos of other species, they were not pluripotent. Even in the mouse, only the 129 strain from which ES cells were originally isolated proved consistently amenable to ES cell derivation and genetic manipulation. However, this strain has the disadvantage of poor breeding efficiency and is seldom the model of choice; multiple costly and time-consuming generations of backcrossing are required to transfer a genetic manipulation from a 129 transgenic to a desired genetic background.

In the late 1990s, pluripotent cell lines were derived from non-human primate and human blastocysts and deemed to be ES cells [8]–[10]. However, they were found to rely upon distinct signaling pathways to be maintained [11]. More recently, human ES (hES) culture conditions were used to isolate cell lines from the *post*-implantation mouse epiblast; these were named epiblast stem cells (EpiSCs) [12], [13]. Primate ES cells and mouse EpiSCs are distinct from mES cells and they share several features that hinder their use with genetic technologies including resistance to single cell dissociation [12]–[14], reduced karyotype stability [15], [16], and limited capacity for chimera formation and germline contribution [12], [13], [17].

**Figure 1:**
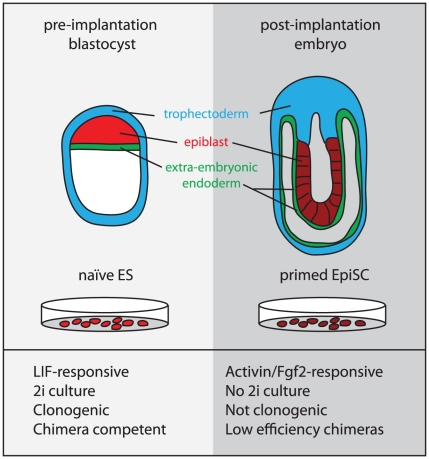
The origin and properties of naïve and primed pluriopotent stem cells.

EpiSCs and mES cells are considered to represent the developmental stages from which they are derived: the post- and the pre-implantation epiblast, respectively (Figure 1) [18]. By the post-implantation stage, random X-inactivation has occurred in the epiblast cells and they are poised to respond to inductive cues at the onset of gastrulation. Likewise, female EpiSCs also harbor an inactive X [19] and may be “primed” towards differentiation as indicated by increased expression of lineage-specific markers [12], [13]. In contrast, in the earlier pre-implantation blastocyst, cells of the epiblast have just been epigenetically “reset.” This is indicated by the reactivation of the paternal X chromosome exclusively in the epiblast cells (of female embryos) [20], [21]. This more “naïve” state appears to be preserved in ES cells, which also harbor two active X chromosomes (when female) and are considered to have an open chromatin conformation [22].

Our limited ability to capture naïve pluripotent stem cells has been a barrier to efficient genetic manipulation in non 129-strain mice and other species. ES cells also have been widely used as a model to study early development and lineage commitment (reviewed in [23]). However, the hitherto limited applicability of ES cell principles across species challenged the relevance of this research to mammalian development in general.

Recently, it has been demonstrated that mES cells can be derived and maintained using small molecule inhibitors of Gsk3 and the Fgf-MAPK signaling cascade (CHIRON99021 and PD0325901, respectively) [24]. This two-inhibitor (2i) culture condition has facilitated the derivation of ES cells from all tested mouse strains [24], [25] and several strains of a second species, *Rattus norvegicus* [26]–[29]. The demonstration that genuine naïve ES cells can be derived from the rat in the same culture conditions suggests that mES cells may indeed represent a common developmental stage, at least in rodents. It has also been reported that hES cell lines can be “reprogrammed” to a state similar to naïve pluripotent mES cells [30], suggesting that this state is more widely conserved across mammals.

## Defining the Requirements for Self-Renewal

Classical culture conditions employed serum-containing media and a layer of mitotically inactivated fibroblasts (feeder) cells [1]–[3]. Initially, little was known about the molecular nature of the self-renewal signals provided by these components. However, in 1988, the key contribution of feeders was determined to be the IL-6 family cytokine LIF [31],[32]. More recently, the anti-neural cytokine BMP4 was found to substitute for serum, and by combining BMP and LIF a defined, feeder-free, serum-free culture condition for ES cell derivation and maintenance was created [33].

The apparent dependence of ES cells upon growth factors underpinned a belief that exogenous signals drive ES cell maintenance. Downstream of LIF, Stat3 was identified as a major functional mediator of self-renewal [34], [35]. The MAPK/Erk pathway was also suspected to be a self-renewal pathway due to its placement downstream of LIF and relatively high activation in ES cells, but was paradoxically found to promote ES cell differentiation [36]. More recently, it has been demonstrated that in serum-free conditions MAPK/Erk activity is driven primarily by autocrine Fgf4 signaling [37]. Importantly, genetic or pharmacological inhibition of the Fgf-MAPK pathway blocks efficient ES cell differentiation [36]–[39]. This finding indicates that shielding ES cells from the inductive signals in their environment is an important aspect for their maintenance in vitro. It has also been observed that early embryos defective in MAPK/Erk signaling [40] or exposed to Mek1/2 [41] or Fgfr [42] inhibitors form an expanded epiblast at the expense of extra-embryonic endoderm, suggesting that the in vitro sensitivity of ES cells to Fgf-MAPK signaling reflects a mechanism of early cell fate decision in vivo. However, Fgf-MAPK pathway inhibition alone is not sufficient for clonal propagation of mES cells. Inhibition of Gsk3 (independently shown to enhance ES cell propagation by pharmacological [43] and genetic [44] means) in addition to Fgf-MAPK restores clonogenicity and permits de novo derivation and long-term propagation of mES cells. Inhibition of Gsk3 up-regulates a broad range of metabolic and biosynthetic processes (reviewed in [45]), and in ES cells, leads to the alleviation of Tcf3-mediated repression of pluripotency factors (J. Wray, T. Kalkan, S. Gomez-Lopez, D. Eckardt, A. Cook, et al, unpublished data). *Stat3*-null ES cells can be derived using Gsk3 and Fgf-MAPK pathway inhibitors (2i), formally proving that extrinsic LIF-STAT3 signaling is not necessary for ES cell self-renewal in these conditions [24]. However, wild-type mES cells cultured in 2i remain sensitive to LIF, exhibiting enhanced cloning efficiency in 2i+LIF as compared to 2i alone [24]. This optimized condition, 2i+LIF, has since been used to derive ES cells from previously recalcitrant strains and species.

## Overcoming Recalcitrance

In 1997, Brook and Gardner noted a “persisting ignorance about the genetic basis of permissivity” [3]. The “permissivity” they referred to was the particular propensity of the 129 strain of mouse for ES cell derivation. The 129 strain was fortuitously used in the first derivations of ES cells due to the historical progression that led from studies of germ line carcinomas to this work [46]. Using conventional serum and feeder conditions, 10%–30% derivation efficiency can be routinely achieved from 129 blastocysts [47], [48]. Brook and Gardner even reported 100% efficiency [3] by deriving from microsurgically isolated epiblasts of implantation-delayed 129 embryos.

While the susceptibility of the 129 strain to ES cell derivation may be related to its propensity to develop gonadal teratomas (tumors with cell types of all three germ layers) [49], the basis of non-129 strain recalcitrance is not fully understood. Strain variation in derivation efficiency and in vitro colony formation appear to correlate with differential Fgf-MAPK signaling [50], [51], but the underlying mechanisms have not been elucidated. Using techniques such as implantation delay, micro-dissection, and inhibitors of Mek1/2 in combination with serum and feeders, ES cell derivation was reported from C57BL/6 [52], DBA/1lacJ [53], CD1 [54], C57BL/6 X CBA [55], PO [3], and CBA and CBA/Ca [3], [50], [56], but efficiencies remained low. Only certain serum batches are suitable for ES cell culture, and the use of serum may have hindered derivation from recalcitrant strains. The development of serum-free media [33] has eliminated this variable and provided a platform for further optimization of ES cell culture and derivation.

Indeed, serum-free 2i culture condition has overcome the limitations imposed by mouse genetic background on ES cell derivation and maintenance. In striking contrast to conventional culture, 2i+LIF yields stable ES cell lines from any tested strain of mouse with high efficiency [24], [25]. Germline-competent ES cell lines have been established not only from 129 mice, but also CBA and MF1 [24] as well as NOD [25] and FVB (J. Nichols, E. Michalak, J. Jonkers, unpublished data). Gene targeting in strains with biologically divergent genetic make-ups and phenotypes is now achievable. Moreover, these culture conditions open up the possibility of generating ES cells from existing transgenics, facilitating efficient combination of targeted mutations.

## Conquering Rodentia

Soon after the first mES cells were derived, work began to isolate ES cells from the rat [57]. As a closely related species and fellow rodent, it seemed a reasonable choice. Furthermore, as the rat is extensively used to study physiology, cognition, and behavior, access to the germline for precise genetic manipulation would be an invaluable tool. However, for 27 years, all attempts were met with failure. Multiple groups, including ours, had reported the derivation of cell lines from rat blastocysts [58]–[62]. But the identity of these lines range from contaminating mES cells [57], [61] to cell lines with properties of extra-embryonic lineages [58], [60]–[63]; none have proved capable of colonizing the germline of chimeras.

However, utilizing 2i, LIF, and fibroblast feeder cells, cells with the morphology and gene expression pattern of ES cells, and most importantly, the ability to contribute to chimeras and make functional germ cells, were derived from the SD [26] and DA [27] strains of rat. This result confirms that a naïve pluripotent cell type can be captured in a species other than the mouse. Other groups have since validated rat ES cell derivation using the two inhibitors to isolate germline-competent cells from several wild-type and transgenic strains [64], [65]. As of yet, no variability in derivation efficiency among strains has been noted, suggesting that ES cells may become available from a multitude of disease modeling rat strains such as the SHR. Notably, both lines reported by Hirabayashi et al. were female [29], as were all six lines derived by Kawamata and Ochiya [28] and all but one line reported by our group [26]. A recent isolation of pluripotent embryonic germ (EG) cells derived from rat primordial germ cells also reported exclusively female lines [66]. While it is apparent that both male and female isolated epiblasts form outgrowths at near 100% efficiency, male lines proliferate more slowly during early passages (K. Blair, unpublished data) and may therefore be selected against early during the derivation process. The underlying cause of this bias against male cells remains unknown, but overcoming it will be important for efficient germline modification.

Qi-Long Ying's group recently reported successful targeting of the *p53* locus in rat ES cell by homologous recombination [67]. This proof of principle report represents a long-awaited advance in the field of rat genetics [68] and mammalian transgenesis, given that the rat is perhaps the most widely used mammalian model with an extensively characterized physiology and behavior [69]. However, this report also highlighted a key difference between mouse and rat ES cells and a potential limitation of rat ES cells as a tool to access the rat germline. While genetic integrity is a hallmark of the mES cell, karyotype stability is a recurrent problem with rat ES cells [26], [27], [70]. Both initial reports indicated karyotypic instability in higher passage cells [26], [27] and initial attempts to transmit the *p53* knockout through the germline failed, with the authors citing >65% polyploidy of the injected line as a likely cause. Only through sub-cloning and careful morphological selection during passaging was a karyotypically normal, germline-competent line maintained [70].

For the full potential of rat ES cells for genetic research to be realized, cell lines and culture methods should be further optimized. It should be remembered that before optimization and standardization of culture conditions throughout the mES cell field, it was widely considered that only early passage cells were germline competent and useful for gene targeting [7], whereas we now know that in the right conditions, mES cells are stable for many generations. Work has begun to optimize rat ES cell culture for stability. Kawamata and Ochiya have reported high germline competence on feeders with their culture media, YPAC [28], a serum-based media with 2i inhibitors, as well as a ROCK inhibitor (shown to reduce dissociation associated apoptosis in hES cells [14]) and an Alk inhibitor (suggested to suppress differentiation of putative rat induced pluripotent stem (iPS) cells [71]). They achieved germline competence from 3/3 lines tested. These results are encouraging, but ideal conditions for culturing ES cells will be those that are highly reproducible among laboratories. Therefore, it is desirable to eliminate undefined, variable components such as serum and feeders. Optimized conditions for the culture of rat ES cells may in turn help us to unlock the naïve pluripotent state in other species, including the human.

## Towards Naïve Human ES Cells

Human and non-human primate ES cells were originally derived by Thomson and colleagues from pre-implantation blastocysts using serum and feeders [8]–[10]. However, despite the similarity of these conditions to those conventionally used to culture mES cells, it has now become clear that these cells have different requirements for self-renewal. While LIF/BMP or 2i can sustain mES cells, Fgf2 and Activin have been identified as the signals that support the maintenance of hES cells [11]. This distinction was initially attributed to species differences. However, more recently, Fgf2/Activin-dependent cell lines were isolated from post-implantation mouse epiblasts [12], [13]. These cell lines were termed epiblast stem cells (EpiSCs) to distinguish them from ES cells. They were also reported to be derived from the rat, though these cells exhibit different Fgf-responsiveness [13]. EpiSCs cannot be derived or maintained in 2i. Furthermore, while ES cells contribute to chimeras with high efficiency, only a small sub-population of EpiSCs under certain culture conditions has this capacity [17]. hES cells are more similar to EpiSCs than they are to mES cells with respect to global gene expression and behavioral characteristics [12], [13], suggesting that hES cells advance developmentally during derivation and that the culture conditions employed tend to capture an EpiSC-like state. However, hES are not identical to mouse EpiSC, expression differences in certain key genes have been identified [72], and the X-inactivation status of female hES seems variable [73], [74]. These differences may be attributable to species divergence in epiblast development [75].

As previously noted, the primed, EpiSC-like state of hES cells is accompanied by characteristics such as low viability after single cell dissociation [12]–[14] and reduced karyotype stability [15], [16] that make them inefficient, though usable, for genetic technology applications [76]. Also, the different developmental states represented by mouse and hES cells dictate that the many established in vitro differentiation protocols developed in the mouse do not translate well to the human system. The derivation of hES cells with characteristics of naïve ES cells would facilitate the transfer of knowledge from model organism to its human equivalent.

EpiSCs can be reprogrammed to an ES cell state by over-expression of the pluripotency transcription factors Nanog [77], Klf4 [19], and Klf2 [78] by Nr5a receptors [79], Stat3 activation [80], or by rare spontaneous reprogramming events [81]. Recently, the Jaenisch group employed reprogramming techniques to establish mES-like human cell lines from fibroblasts and existing hES cells [30]. They established lines in 2i+LIF that exhibited an epigenetic and gene expression profile similar to mES cells. Like both mouse and rat ES cells, but unlike established hES cells, they responded functionally to LIF. However, these naïve hES cell lines were dependent on continued expression of the reprogramming transgenes. The addition of forskolin to the media allowed the establishment of naïve hES cell lines that were not dependent on transgene expression but these lines could not be maintained for more than 20 passages. Also using a reprogramming approach, Buecker et al. succeeded in establishing LIF-responsive hES cell lines [82]. The authors claimed these cells were more amenable to genetic manipulation than lines maintained under standard hES culture conditions. Together, these studies suggest the feasibility of generating naïve hES cells and highlight their potential as a tool for genetic modification. Future work will determine whether conditions can be established that support the derivation and long-term propagation of naïve hES cells direct from the human embryo.

## Conclusions

Advances in our understanding of ES cell biology in the mouse have led to the development of a culture condition for self-renewal based on small molecule inhibitors of Fgf-MAPK signaling and Gsk3. Employing these inhibitors, naïve pluripotent ES cells have been derived from multiple strains of mouse and rat and are presenting new opportunities for genetic intervention in these species. It is plausible that since rodents have a capacity for developmental suspension at the blastocyst stage, a phenomenon known as diapause, that the ability to capture the naïve ES cell state may be specific to the limited number of species with this ability (discussed in [18]). However, until recently this cell type was in practice a 129 mouse-specific phenomenon. Only by expanding our knowledge of ES cell biology and developing defined conditions have we succeeded in capturing and culturing ES cells from multiple mouse strains, the rat, and through genetic intervention, the human. These recent advances suggest that the naïve pluripotent state may be conserved across species but that we have lacked the means to capture it in vitro from hitherto recalcitrant mammals. While this question primarily relates to developmental stem cell biology, its resolution has consequences for the study of genetics. We have already seen that the genomes of genetically diverse mouse strains are easily accessed by 2i culture. The unparalleled resource that naïve ES cells present for genetic manipulation would be an invaluable tool if it could be readily applied to a wider range of species. If we could manipulate the genome of cultured human cells with the facility already achieved in the mouse, this would represent a boon for developmental and disease modeling in vitro. For many applications, including livestock improvement or species conservation, developing a widened array of ES or iPS cells from diverse species promises new opportunities.
